# Light and Low Relative Humidity Increase Antioxidants Content in Mung Bean (*Vigna radiata* L.) Sprouts

**DOI:** 10.3390/plants9091093

**Published:** 2020-08-25

**Authors:** Chiara Amitrano, Carmen Arena, Stefania De Pascale, Veronica De Micco

**Affiliations:** 1 Department of Agricultural Sciences, University of Naples Federico II, 80055 Naples, Italy; chiara.amitrano@unina.it (C.A.); depascal@unina.it (S.D.P.); 2 Department of Biology, University of Naples Federico II, 80126 Napoli, Italy; carena@unina.it

**Keywords:** air relative humidity, antioxidants, controlled environment agriculture (CEA), morpho-anatomical traits, light

## Abstract

In the last decades, there has been a growing interest in the production of sprouts, since they are a highly nutritious food, particularly suitable for indoor farming in urban areas. Achieving sprout production in indoor systems requires an understanding of possible alterations induced by the microclimate. The aim of this study was to analyze the combined effect of presence/absence of light and high/low air relative humidity (RH) on mung bean sprouts. Morpho-anatomical development and functional anatomical traits in hypocotyl were quantified. The content of antioxidants, soluble sugars, and starch were measured for nutritional and functional purposes. Different RH regimes mainly induced morpho-anatomical modifications, while the presence/absence of light changed the content of antioxidant compounds. Increments in stele diameter at high RH suggest a higher water uptake and conductivity, compared to the low RH treatment; low RH and light induced anatomical traits improving plant water transport (reduced number of cortical layers) and increased the production of antioxidants. The overall results suggested that RH and light, already at the early stages of development, affect the plant’s nutritional value. Therefore, the combination of light and low RH allows the production of antioxidant-rich mung bean sprouts to be used as a food supplement.

## 1. Introduction

Agricultural practices are changing with the introduction of new technologies for the remote monitoring and controlling of environmental conditions, such as intelligent sensors, robots, proximal/remote sensing [1,2]. These new technologies applied to agriculture are helping to meet the nutritional requirements of an increasing population, minimizing environmental impacts and coping with the scarcity of resource availability, also exacerbated by climate changes [3]. Part of this change is happening in urban areas, where over 54% of the world population is concentrated [4] and which consumes most of the world’s energy and resources while producing the majority of greenhouse gases [5]. In this context, controlled environment agriculture (CEA) and, more specifically, urban farming (e.g., rooftop greenhouses, indoor-growing modules) is receiving increasing attention as a method to improve food security, enhance sustainability, and contribute to saving transportation and logistics costs [4,5]. Currently, we are witnessing a growing interest in urban agriculture, which has been well documented by backyard food production, local-sustainable-organic restaurants, and the rise of plant-based-diets, suggesting that people desire to “get closer to their food” [6,7,8]. Plant-derived fresh food not only has high nutritional value but also plays a role as a stress mitigator for citizens who are subjected to urbanicity effects [9,10,11]. However, the possibility of growing edible crops as sources of fresh and high nutritional food directly in houses and restaurants relies on technological advancements in CEA, as well as on the development of cultivation protocols to increase crop yield while enhancing the nutritional properties of edible plant parts. In this context, sprouts and microgreens have recently gained popularity as easy-to-produce (e.g., small size, without soil and external inputs as fertilizers and pesticides), healthy gastronomic ingredients [12,13]. Indeed, compared to mature-leaf crops, they contain a higher amount of bioactive compounds, antioxidants, minerals, and phytonutrients [14]. Their consumption has been associated with a reduced risk of cancer, respiratory problems, osteoporosis, and muscle atrophy, which are frequent diseases associated with obesity and malnutrition [15,16,17]. Among these, *Vigna radiata* L. (mung bean) presents a high nutritional value of its sprouts, whose consumption has been related to antioxidant, anti-inflammatory, antidiabetic, antihypertensive, and antitumor effects [18,19]. Significant challenges in indoor agriculture, include the control of relative humidity (RH) and the demand for power supply, needed to artificially maintain a precise microclimate and especially light regimes to grow different plant species [20,21,22]. Through humidity management, it is possible to reduce water footprint, influencing the rates of water loss by crop transpiration. In addition to saving water, humidity control is important for crop performance because it influences many physiological processes from germination to senescence, such as cell expansion, photosynthesis, and growth [23,24]. Although RH control in CEA has often been overlooked due to technical constraints [23], modulation of lighting systems and different RH regimes can increase crop growth performance and enhance food nutritional properties [25].

Sprouts production partly overcomes these problems because they require small production facilities, shallow substrate, and very short time (4–8 days) to reach a commercial edible size [26]. Furthermore, sprouts cultivation needs low or no demand for photon flux: indeed, most seeds do not require light for sprouting and they might even be inhibited by light (e.g., *Phacelia* and *Allium*). However, other species (e.g., *Begonia*, *Primula*) need light to germinate [27]. Generally, sprouts used as food-supplement are grown in total darkness and under high levels of relative humidity, to reduce the risk for drying between watering periods [28].

Even though sprouts are usually grown etiolated, lighting systems in indoor growing modules can be modulated to achieve compound-specific improvements in sprouts’ quality and sometimes to decrease the level of anti-nutrients [13], especially during the phase of seed germination [29]. Even though lightening systems can increase production costs [21], changes during the early stages of germination lead to increments in some useful metabolites such as flavonoids, phenols, vitamins and other phytochemicals [30,31]. Many studies have tested the effect of light intensity and quality on sprout phytochemical content, with contrasting results depending on the PPFD (photosynthetic photon flux density) and on the specific wavelengths [18,32,33]. However, the general tendency is to have a higher quantity of antioxidants in light-grown sprouts compared to dark-grown ones [34,35,36,37], even if sprouts grown in the light can be smaller compared to those grown in the dark [38].

Concerning the implications of RH on sprout production, several authors have demonstrated that increasing humidity during the cultivation could enhance quality traits. For example, Leyva et al. [39] found an increment in antioxidant content in many tomato cultivars grown in the presence of fogging systems. However, cultivation under different RH regimes causes modifications in the organization of crop tissues, cell size, and density [40,41], which may, in turn, affect physiological mechanisms, resource use, and tissue/organ allocation of bioactive compounds.

So far, most of the research has been conducted on adult plants, while only a few studies have been performed aiming to control RH and other environmental factors to enhance sprout growth and phytochemical production. However, the optimization of cultivation protocols already at the first stages of plant development is required not only to optimize sprout growth and enhance the production of useful secondary metabolites but also to achieve fine control of environmental parameters for sustainability issues (i.e., energy consumption). The aim of this study was to analyze the combined influence of two environmental factors namely presence/absence of light and high/low RH, on morpho-anatomical development and production of nutritious secondary metabolites in sprouts of *V. radiata* L. “green azuki”, a plant species in the legume family, also known as mung bean. For these reasons, we evaluated sprouts development through morpho-anatomical analyses and their antioxidant content (i.e., anthocyanins and polyphenols) as well as the amount of soluble sugars and starch, considered as important nutritional traits.

## 2. Results

### 2.1. Sprouts Development

The total length of sprouts, the length of roots and hypocotyls as well as the fresh weight were influenced by RH and LR as main factors, but not by their interaction (RH × LR) (Table 1). Both length and fresh weight significantly (*p* ≤ 0.001) increased under high RH, independently from the presence/absence of light. Moreover, sprouts grown in the dark were always significantly more elongated (*p* ≤ 0.001) and presented a higher fresh biomass (*p* ≤ 0.05) compared to those grown under light. Conversely, dry weight was neither significantly influenced by RH and LR as main factors, nor by their interaction (Table 1).

### 2.2. Morpho-Anatomical Analysis of Hypocotyls

**Figure 1:**
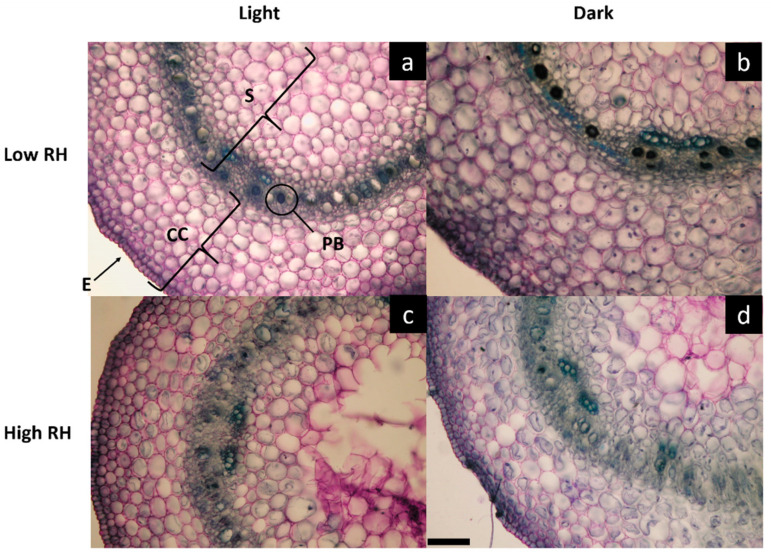
Light microscopy views of cross-sections of hypocotyls of mung bean sprouts grown under the four different combinations of RH and LR: (**a**) LowRH-L; (**b**) LowRH-D; (**c**) HighRH-L; (**d**) HighRH-D, High RH in dark regime). Stele (S), Cortical cylinder (CC), Epidermis, (E) and Phenolic bodies (PB). Images are at the same magnification; scale bar = 100 µm.

Microscopy analysis evidenced that the hypocotyls maintained tissue integrity, without evident signs of stress in all the combinations of RH and LR (Figure 1). However, the expansion of the cortical cylinder (in terms of thickness) and stele (in terms of diameter) were significantly influenced by both RH and LR treatments as main factors as well as by their interaction (RH × LR) (Table 2). The sprouts grown in the dark at the lower relative humidity (LowRH-D) were characterized by the widest cortical cylinder (TCC), which was significantly thicker (*p* ≤ 0.05) compared to the other conditions (Table 2). As regards the stele, the smallest diameter (SD) was found in LowRH-L sprouts, with significantly lower values (*p* ≤ 0.01) compared to all the other treatments (Table 2). Moreover, the number of cell layers in the cortical cylinder (CL-CC) was not influenced by RH as the main factor; however, light alone and in interaction with RH, had a significant effect on CL-CC (*p* ≤ 0.05). Conversely, no significant differences among treatments were found in the number of cell layers in the stele (CL-S). Furthermore, the number of cells per unit area in both cortical cylinder and stele (NC-CC and NC-S) were significantly influenced (*p* ≤ 0.001 and *p* ≤ 0.05 respectively) by microclimate interaction (RH × LR) (Table 2 and Figure 1). Both NC-CC and NC-S were significantly higher in LowRH-D than HighRH-L sprouts, which showed in turn significantly higher values than sprouts developed under the other two conditions. The number of phenolic bodies (NPB) was significantly influenced by both RH and LR treatments as main factors (*p* ≤ 0.001) as well as by their interaction (RH × LR) (*p* ≤ 0.05) (Table 2). Moreover, the diameter of phenolic bodies (DPB) was significantly influenced by LR treatment as main factor (*p* ≤ 0.001) and by the interaction (RH × LR) (*p* ≤ 0.01) (Table 2). More specifically, LowRH-L presented the highest values of both NPB and DPB, which instead were significantly reduced in HighRH-D.

### 2.3. Content of Anthocyanins and Polyphenols, and FRAP Assay

**Figure 2:**
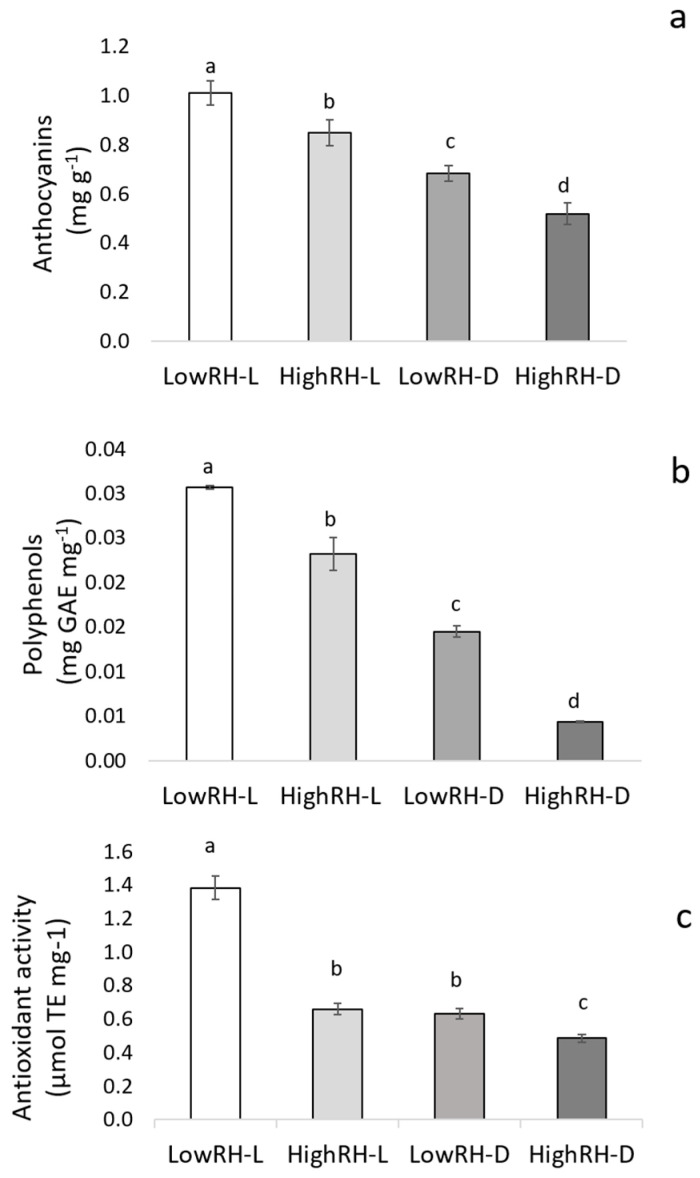
Concentration of anthocyanins (**a**), polyphenols (**b**) and antioxidant activity (**c**) in mung bean sprouts grown under the four different combinations of RH and LR (LowRH-L; LowRH-D; HighRH-L; HighRH-D). Mean values and standard errors are reported; different letters refer to statistically significant differences; *p* ≤ 0.05).

For anthocyanins and polyphenols, both RH and LR as main factors (*p* ≤ 0.001) and their interaction (RH × LR) (*p* ≤ 0.05) elicited significant changes (Table 3). The antioxidant activity was significantly influenced by both RH and LR as main factors (*p* ≤ 0.001) and their interaction (RH × LR) (*p* ≤ 0.001), as well (Table 3). The concentration of anthocyanins and polyphenols, as well as an antioxidant activity are shown in Figure 2. The highest content of polyphenols and anthocyanins occurred in sprouts grown at low RH in the presence of light (LowRH-L); such contents significantly decreased in HighRH-L sprouts, which in turn showed significantly higher values than LowRH-D (*p* ≤ 0.05) (Figure 2a,b). Finally, LowRH-D sprouts showed significantly higher values than HighRH-D ones where the lowest contents were detected (Figure 2a,b). The antioxidant capacity showed the same trends among treatments. The highest antioxidant activity occurred in LowRH-L sprouts, which showed significantly higher values than HIghRH-L and LowRH-D sprouts (*p* ≤ 0.001) (Figure 2c). The lowest values, significantly reduced compared to all the other treatments, were detected in HighRH-D sprouts (Figure 2c). The content of anthocyanins and polyphenols were positively correlated with antioxidant capacity (r = 0.757, *p* ≤ 0.001 for anthocyanins and antioxidant capacity and r = 0.821, *p* ≤ 0.001 for polyphenols and antioxidant capacity).

### 2.4. Soluble Sugar and Starch Quantification

**Figure 3:**
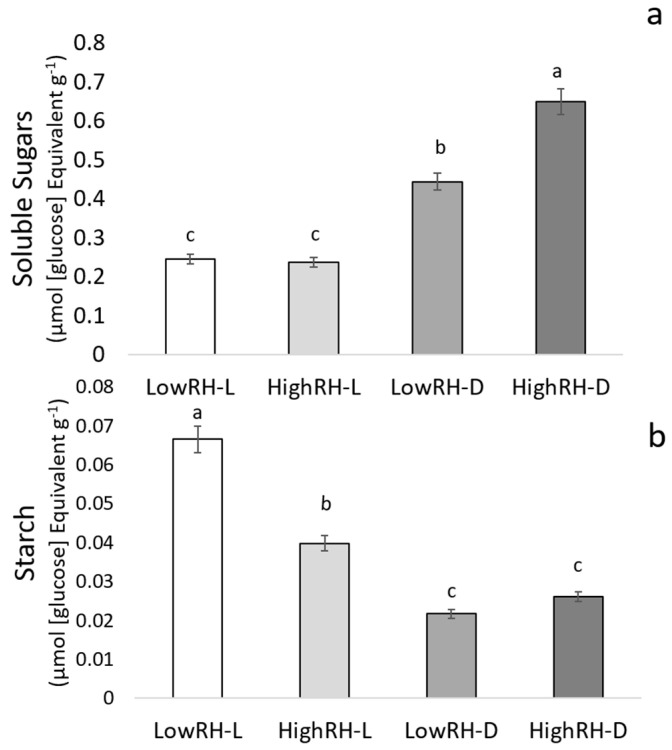
Soluble sugar (**a**) and starch (**b**) amount in mung bean sprouts grown under the four different combinations of RH and LR (LowRH-L; LowRH-D; HighRH-L; HighRH-D). Both soluble sugars and starch are expressed on a fresh weight basis. Mean values and standard errors are reported; different letters refer to statistically significant differences; *p* ≤ 0.05.

The content of soluble sugars was significantly influenced by RH and LR as main factors (*p* ≤ 0.001 and *p* ≤ 0.01 respectively), and by their interaction (RH × LR) (*p* ≤ 0.01) (Table 3). Starch content was significantly influenced by both RH and LR as main factors (*p* ≤ 0.05), and by their interaction (RH × LR) (*p* ≤ 0.05) as well (Table 3). Their content showed an opposite trend among treatments (Table 3 and Figure 3). More specifically, HighRH-D sprouts showed significantly higher values of soluble sugars than LowRH-D sprouts (*p* ≤ 0.01), which, in turn, were characterized by significantly higher values compared to sprouts developed in the presence of light, independently from humidity (Figure 3a). Regarding starch content, sprouts grown under LowRH-L conditions showed significantly higher values than HighRH-L sprouts (*p* ≤ 0.05), which, in turn, were characterized by significantly higher values than sprouts developed in the dark, independently from humidity levels (Figure 3b).

## 3. Discussion

During seed germination, complex biochemical and physiological processes occur, resulting in wide changes in sprout morphology and biochemical composition [42]. This process is triggered by the imbibition of water and is controlled by the whole complexity of environmental parameters [43]. In the present study, the two main factors applied during sprouting (RH and LR), had significant effects on mung bean sprouts growth, morpho-anatomical development, antioxidant capacity, amount of soluble sugars and starch. More specifically, the level of humidity under which sprouts were grown, mostly influenced their morphological development. Indeed, the increase in RH from 60% to 90% lead to sprout lengthening. Furthermore, this elongation was not followed by any increment in total hypocotyl diameter, as well as in the number of cell layers in the cross-section. These results suggest that increased elongation at high RH was either due to enhanced apex proliferation or turgor driven cell-enlargement in the longitudinal direction. The humidity-driven increase in fresh weight (more than doubled under high RH compared to low RH) supports the second hypothesis. The higher turgor-driven cell enlargement is also consistent with the observed lower number of cells per stele unit area, which could be explained by an increase in cell size and unaltered dry weight among treatments. Similar results were found by McIntyre et al. [44] in the apex growth of potato tubers; these authors hypothesized that high humidity may have increased water potential and cell turgor of sprouts, due to the reduction in their rate of transpiration.

In mung bean, the interaction among factors (RH and LR) elicited changes in quantitative anatomical traits in terms of the relative ratio between cortical cylinder and stele, and the number of cells per unit area (thus consequently in cell size) in both tissues (Table 2). The differences in hypocotyl anatomical traits could be the result of adjustment strategies to different environmental conditions. For instance, the occurrence of decreased root diameters under reduced water availability is considered a way to increase water and nutrient uptake by maximizing the absorptive surfaces [45,46]. Furthermore, the reduced number of cortical layers is known to be an acclimation to drought; indeed, under drought the reduced number of cell layers shortens the radial pathway available for water transport, favoring a quick radial flow [47,48]. These results highlight the pivotal role of anatomy in controlling water movement through the soil-plant system and the relationship between xylem anatomy and hydraulic conductivity [49]. Two of the major anatomical features that distinguished categories of root anatomy are indeed the stele diameter and the arrangement of the cortical cells [50,51]. Changes in the size of the cortical cylinder and not in that of the stele has been explained by assuming the cortex acting as a “buffer zone”, partially isolating the stele from environmental stresses such as drought [52,53]. Our results are in agreement with the tendency of having a larger “buffer zone” under conditions of low humidity.

The presence of light, and more specifically its intensity and quality, acts on sprout antioxidant content. In the last decades, many studies have dealt with the effects of lighting on crop quality, with very different results depending on photosynthetic photon flux density (PPFD) or light wavelength quality. Although sprouts are commonly produced in the absence of light, it has been demonstrated that even a low amount of PPFD may induce positive outcomes during germination. For example, Pérez-Balibrea et al. [34] found in light-grown broccoli sprouts an increment in phenolic compounds (by 61%) compared to sprouts grown in the dark. In contrast, Qian et al. [54] found in Chinese kale sprouts grown under blue-light, the highest levels of total phenolics and anthocyanins, as well as the strongest antioxidant capacity. Phenolics are the products of secondary plant metabolism, which provide essential functions in growth and reproduction [55]. Epidemiological and experimental studies demonstrated that phenolic compounds in the human diet may provide health benefits associated with reduced risk of chronic diseases [56]. In our study, the presence of light enhanced the content of both anthocyanins and phenolics, as also visualized through microscopy (i.e., phenolic bodies), especially under low-humidity growing sprouts. These sprouts (LowRH-L), which exhibited the smallest length, also presented the highest antioxidant capacity. It is known that light improves the phenolic content by promoting the production of malonyl CoA and coumaroyl CoA, which participate in the synthesis of phenolic compounds [57]. Therefore, it is not surprising that, in our study, a significant increase in total phenolics was found in light-grown sprouts compared to the dark ones. Furthermore, antioxidants and polyphenols are often synthesized by plants as a defense mechanism in response to abiotic and biotic stresses [58,59,60]. Under natural conditions, plants grown in semi-arid climate enhance the production of secondary metabolites to counteract the exposure to high levels of solar radiation, high temperature, and low water availability [61]. The secondary metabolite products may contribute to preventing damages caused by reactive oxygen species (ROS) during the environmental stresses. This is consistent with the increase in the levels of polyphenols and anthocyanins in sprouts grown at low RH, notwithstanding the light regime. Moreover, many studies have reported an enhanced content of polyphenols and anthocyanins in sprouts or microgreens cultivated under light [13,35,62]. Indeed, the exposure to light may be considered the key stimulus for the synthesis of anthocyanins [32,63]. Consistently, in our experiment, the light regime increased the anthocyanin content in mung bean sprouts, which in turn resulted in a bright red color of the hypocotyls.

In addition, we found a high correlation between the variation of total polyphenols and anthocyanins, and the antioxidant capacity. Antioxidant capacity, which gradually increases during germination [55] is an important quality index reflecting the synergetic effect of multiple antioxidants, including phenolic compounds. A similar tendency was also found by Gan et al. [64] in a study on mung bean sprouts, where phenolic compounds and ascorbic acid increased along with increments in antioxidant capacity. Indeed, phenolic compounds have been proved to contribute more than other antioxidants to the antioxidant capacity [65]. Moreover, Qian et al. [54] observed a significant increment in the antioxidant capacity in sprouts grown under different light treatments compared to dark, in accordance with the variation tendency of anthocyanin content. In the very early stages of development, the presence of light, determines a rise in antioxidant compounds, together with the onset of photosynthetic activity. Furthermore, it is known that the germination process leads to catabolism and degradation of main storage compounds, often accompanied by an increase in simple sugars, which are an important energy source for seeds during germination and early growth of plants [64,66]. However, the accumulation of sugar can change with environmental conditions or can be subjected to a different allocation [67]. For example, Gill et al. [68] found in seedlings of *Sorghum bicolor* L. grown in the presence of light, an elevated content of soluble sugars in comparison to dark-grown seedlings, especially under stressful conditions (Heat, Cold, NaCl treatments).

However, in our study, soluble sugars content significantly decreased in sprouts grown in the presence of light. This could be explained by a simultaneous increase in the synthesis of starch. It is well known that light and consequently, day length and circadian rhythm have a significant effect on starch degradation and synthesis. It has demonstrated that starch breakdown was faster in plants growing in long days and during the night [69].

In conclusion, the overall results suggested that *V. radiata* sprouts are largely responsive to changes in environmental conditions in terms of tissue development, biomass allocation, and antioxidant production. Our results indicated that under light and low RH sprouts have evolved morpho-functional traits to cope with low RH conditions as a mechanism to elude water loss in the very early stages of development. Furthermore, sprouts grown in the presence of light, especially at low RH, increased polyphenols and anthocyanins contents as well as improved antioxidant capacity, compared to dark conditions. Synthesis and accumulation levels of antioxidant compounds mostly depend on genotype; however, they are also largely affected by environmental factors (microclimate). Therefore, by manipulating the environment, it is possible to obtain antioxidant-rich sprouts with health-promoting properties for consumers [70,71]. In our study, the combination of light-low RH was the most effective for the production of antioxidant-rich mung bean sprouts to be used as a food supplement. This could open new market opportunities in the niche of healthier and vegan food [72].

The main outcome of this study is that a fine control of all environmental variables already at the early stages of plant development should be a priority not only for optimizing plant growth but also for favoring the synthesis of useful metabolites in controlled environment agriculture production.

## 4. Materials and Methods

### 4.1. Experimental Design and Plant Material

The experiment was conducted on *V. radiata* L. by placing 120 seeds in Petri dishes (30 seeds per petri dish), layered with filter paper, and imbibed with distilled water till paper capacity. The experiment was performed in three replicates. *V. radiata* seeds were purchased from a local retailer and showed 100% germination. Petri dishes were incubated, open, in two cycles, in a multi-layers walk-in climatic room at the Department of Biology of the University of Naples Federico II (General Impianti S.A.S., Naples, Italy), at a fixed temperature of 23 ± 2 °C, under four combinations of two relative humidity (RH) and light regimes (LR) at the sprouts level: (a) 60 ± 2% RH, 1.2 kPa (Vapour Pressure Deficit; VPD), 150 ± 20 μmol photons m^−2^ s^−1^ in the Photosynthetic Active Radiation (PAR) region (LowRH-L); (b) 60 ± 2% RH, in the dark (LowRH-D); (c) 90 ± 2% RH, 0.3 kPa (VPD), 150 ± 20 μmol photons m^−2^ s^−1^ in the PAR region (HighRH-L); (d) 90 ± 2% RH, in the dark (HighRH-D). The chosen RH treatments were considered the minimum and maximum levels of the RH range to be maintained to avoid water stress or the spreading of plant diseases, respectively [39]. In LowRH-L and HighRH-L treatments, light was provided by white fluorescent tubes (Sylvania luxline plus-T8, F36W/840, Cool white deluxe, Germany), with a 12 h photoperiod (8 a.m.–8 p.m.) which resulted in a 6.4 DLI (daily light integral), considered low light intensity for micro-scale vegetable production [13,28]. VPD values were calculated from the corresponding instantaneous T and RH values monitored by mini-sensors (Testo 174H, Testo, Germany) and sampled every 15 min. The indoor air temperature and air-current speed were controlled by a heat pump-based air conditioning system with three inlet vents and one outlet vent on the same side wall; whereas, RH was maintained at 60% by means of a dehumidifier (ADH-1000, Airrex Portable dehumidifier, Hephzibah Co. Ltd., Nam-gu, Incheon, Korea). Seedlings were watered every day with fresh distilled water up to water holding capacity of the paper) and harvested after 8 days, before the appearance of the first two leaves, when they reached a commercial edible size standard marketable size, according to Lal and Shanmugasundaram [26]. At the end of the growth period, the percentage of germination was detected and the growth of the 90 sprouts per each treatment was quantified by measuring epicotyl, hypocotyl and root elongation on digital images (taken during the light hours) through ImageJ 1.45 software (U.S. National Institutes of Health, Bethesda, MD, USA). The fresh weight (FW) and the dry weight (DW) were measured on 30 sprouts per treatment (10 sprouts per replicate). Once tested the homogeneity of the samples, biochemical and morpho-anatomical analyses were performed on 6 and 5 sprouts per treatment, respectively, randomly taken. All these analyses were carried out considering the single sprout as one replicate. For the biochemical assays, the sampling was carried out at 9:00 a.m. in the morning.

### 4.2. Morpho-Anatomical Analyses

Hypocotyls from the six sprouts per treatment were dissected under a reflected light microscope (SZX16; Olympus, Germany) and immediately fixed in F.A.A. (5 mL 40% formaldehyde, 5 mL glacial acetic acid, 90 mL 50% ethanol) for several days. The hypocotyl region was chosen for the analyses because the sprout nutritional quality is mainly influenced by hypocotyl length and anatomical traits. Moreover, in sprout production for human consumption, the main aim is to produce germinated seedlings which have not yet developed true leaves; thus, the primary interest was focused on the hypocotyl region [27]. Thin cross-sections (5 µm thick) of hypocotyls were cut by means of a sliding microtome.

The sections were stained with 0.5% Toluidine blue in water [73], mounted with mineral oil for microscopy, and observed under a light microscope (BX60; Olympus, Hamburg, Germany). Digital images were captured using a digital camera (XC50; Olympus) and were analyzed through an image analysis software (AnalySIS 3.2, Olympus, Hamburg, Germany) to quantify anatomical traits. More specifically, the following traits were measured (in four replicates per section): the thickness of cortical cylinder (TCC), stele diameter (SD), total diameter (TD), calculated as the sum of TCC and SD, number of cell layers in the cortical cylinder (CL-CC) and stele (CL-S), and number of cells per unit area in the cortical cylinder (NC-CC) and stele (NC-S). Furthermore, on the same thin cross-sections, phenolic bodies were detected because they appeared colored in dark blue [74]. The presence of phenolic compounds was quantified in terms of the number of phenolic bodies per unit area (NPB) (in four areas per section) and the diameter of phenolic bodies (DPB) (in 3–7 phenolic bodies per section). We also checked for the presence of abnormal lignification, which could have affected the taste and texture of sprouts grown under the light regime, through epi-fluorescence microscopy (BX60, Olympus) with specific settings (Mercury lamp, band-pass filter 330–385 nm, dichromatic mirror 400 nm and above, barrier filter 420 nm and above) for the detection of the UV-induced fluorescence of lignin and other phenolic compounds [75,76,77]. Under such settings, lignin emits light blue fluorescence, suberin produces white-violet fluorescence, and simple phenolics (those stained in dark blue with Toluidine blue) appear yellow-orange fluorescent. Since no abnormal phenomena of either lignification nor suberization were detected, we decided to quantify only the content of polyphenols and anthocyanins through biochemical assays.

### 4.3. Polyphenol and Anthocyanin Content

Both polyphenols and anthocyanins were determined spectrophotometrically on each of the five sprouts per condition. For polyphenols determination, the procedure reported by Singelton and Rossi [78], modified by Costanzo et al. [79], was used. Briefly, 200 mg of the fresh sample from each hypocotyl were ground in methanol at 4 °C and then centrifuged at 11,000 rpm for 5 min. Pellets were discarded, while supernatants were mixed with 1:1 (*v*/*v*) 10% Folin Ciocâlteu and 1:5 (*v*/*v*) 700 mM Na_2_CO_3_ solution. Samples were then incubated at 4 °C for 2 h. The absorbance was determined at 765 nm (Cary 100 UV-VIS, Agilent Technologies, Santa Clara, CA, USA) and the concentration was expressed as gallic acid equivalents (GAE mg mg^−1^) using the regression equation between the different concentration of gallic acid standard and the absorbance at 765 nm. Anthocyanin levels were determined following Neff and Chory [80], by incubating the sprouts overnight in 150 mL of methanol, acidified with 1% HCl. Anthocyanins were determined by measuring the absorbance at 530 and 657 nm of the aqueous phase. More specifically, anthocyanins were calculated by subtracting one-fourth of the absorbance at 657 nm from the absorbance at 530 [81], to take into account the overlapping with chlorophylls whose increase in absorbance is different at the two wavelengths when in acidic methanol solution.

Therefore, the relative amount of anthocyanins per seedling (mg g^−1^) was calculated as follows (Equation (1)):

Acy = (A 530 − (0.25 ∗ A 657)) ∗ extraction volume (mL)/1000 ∗ g (FW)(1)

### 4.4. FRAP Assay

The antioxidant capacity was evaluated through the Ferric reducing/antioxidant power (FRAP) assay on each of the five sprouts per condition, following the procedure reported in George et al. [82], modified by Motta et al. [83]: 250 mg of the fresh sample from each hypocotyl were ground in liquid nitrogen and treated with methanol/water (60/40, *v*/*v*) solution. Samples were then centrifuged, and the supernatant was collected for the assay. The assay was carried out by adding 2.5 mL of acetate buffer, pH 3.6, 0.25 mL of TPTZ (4,6-tripyridyl-s-triazine, Fluka Chemicals, Buchs, Switzerland) solution (10 mM) in 40 mM HCl, 0.25 mL of FeCl_3_·6H_2_O solution (12 mM), and 150 mL of the supernatant obtained from the above extraction.

After 30 min of incubation at room temperature, the absorbance of the formed product (ferrous tripyridyl triazine complex) was read spectrophotometrically at 593 nm. Results were expressed as micromoles of Trolox equivalents (TE) per mg, obtained from the standard curve between 20 and 800 µM Trolox.

### 4.5. Soluble Sugar and Starch Quantification

Soluble sugar and starch were extracted from each of the five sprouts per condition and quantified following the anthrone method [84]. Briefly, 100 mg of the fresh sample from each hypocotyl were firstly ground to powder, then sugars were extracted in 2.5 N HCl and their concentration was determined by the anthrone reaction (ACS reagent 97%, Sigma–Aldrich, Saint Louis, MI, USA) and sulfuric acid. When anthrone reacts with carbohydrates, in a hot bath, a green-colored product emerges. Its absorbance was read spectrophotometrically at 630 nm. Once sugars suspended in the supernatant were removed and analyzed, pellets were evaporated to dryness and used for starch extraction by means of perchloric acid solution. In hot acidic medium, starch is hydrolyzed to glucose and dehydrated to hydroxymethyl furfural. Subsequently, a standard curve with different glucose concentrations was prepared, and results were expressed as µmol glucose equivalent g^−1^. For starch concentration, the value obtained from the standard curve was multiplied for 0.9, as 0.9 g of starch yield as 1 g of glucose on hydrolysis [85,86].

### 4.6. Statistical Analysis

Results were subjected to statistical analysis using SPSS^®^ statistical software (SPSS, Chicago, IL, USA). The influence of the two different categorical independent factors (i.e., relative humidity, RH − 2; light regimes, LR − 2), and their possible interaction, on each of the continuous dependent variables were studied by applying two-way analysis of variance (ANOVA). In the case of rejection of the null hypothesis, the Duncan and Student–Newman–Keuls (SNK) post-hoc tests were used (*p* ≤ 0.05). Whenever the interaction between RH and LR was significant, data were subjected to one-way ANOVA and multiple comparison tests were performed with Duncan and SNK coefficients, considering as significant level of probability *p* ≤ 0.05. The Kolmogorov–Smirnov and Shapiro–Wilk tests were performed to check for normality and a Levene’s test of homogeneity was used to determine if samples had equal variance. Finally, to check for correlations among anthocyanins, polyphenols, and FRAP assay, a Pearson rank correlation coefficient was calculated.

## Figures and Tables

**Table 1:** Analysis of variance and means comparison for growth traits in mung bean sprouts under low and high RH and light or dark LR as well as under the four different combinations of RH and LR (LowRH-L; LowRH-D; HighRH-L; HighRH-D). Mean values and statistical significance are shown. NS, *; **, *** Non significant or significant at *p* ≤ 0.05, 0.01, and 0.001, respectively. Different letters within each column indicate significant differences, according to Duncan’s multiple-range test (*p* ≤ 0.05).

Treatments	Hypocotyl Length (cm)	Root Length (cm)	Total Length (cm)	Fresh Weight (g)	Dry Weight (g)
Relative humidity (RH)					
Low	2.53 b	0.88 b	3.74 b	0.22 b	0.044 a
High	3.47 a	1.93 a	3.95 a	0.46 a	0.044 a
Light regime (LR)					
Light	2.69 b	1.18 b	3.55 b	0.32 b	0.048 a
Dark	3.32a	1.62 a	4.13 a	0.37 a	0.046 a
Interaction					
LowRH × light	2.26 a	0.63 a	3.40 a	0.20 a	0.047 a
HighRH × light	3.12 a	1.73 a	3.71 a	0.44 a	0.049 a
LowRH × dark	2.80 a	1.13 a	4.08 a	0.25 a	0.042 a
HighRH × dark	3.83 a	2.12 a	4.19 a	0.49 a	0.042 a
Significance					
RH	***	***	***	***	NS
LR	***	***	***	*	NS
RH × LR	NS	NS	NS	NS	NS

**Table 2:** Analysis of variance and means comparison for the thickness of cortycal cylinder (TCC), stele diameter (SD), total diameter (TD), number of cell layers in the cortical cylinder (CL-CC) and in the stele (CL-S), number of cells per unit area in the cortical cylinder (NC-CC), and stele (NC-S), number of phenolic bodies (NPB), Diameter of phenolic bodies (DPB) in mung bean sprouts under low and high RH and light or dark LR as well as under the four different combinations of RH and LR (LowRH-l; LowRH-D; HighRH-L; HighRH-D). Mean values and statistical significance are shown. NS, *; **, *** Non significant or significant at *p* ≤ 0.05, 0.01, and 0.001, respectively. Different letters within each column indicate significant differences, according to Duncan’s multiple-range test (*p* = 0.05).

Treatments	TCC (µm)	SD (µm)	TD (µm)	CL-CC (n)	CL-S (n)	NC-CC (n mm^−2^)	NC-S (n mm^−2^)	NPB (n mm^−2^)	DPB (µm)
Relative humidity (RH)									
Low	963.75 a	1418.13 b	2381.88 a	10.05 a	16.37 a	25.35 a	21.10 a	40.07 a	4.0 a
High	817.14 b	1554.28 a	2371.43 a	10.02 a	17.82 a	23.77 a	18.02 b	27.54 b	3.75 a
Light regime (LR)									
Light	828.46 b	1429.33 b	2257.80 b	9.8 b	17.45 a	29.67 a	23.10 a	40.76 a	4.67 a
Dark	952.43 a	1543.07 a	2495.51 a	10.27 a	16.75 a	19.45 b	16.02 b	26.85 b	3.07 b
Interaction (RH × LR)									
LowRH × light	886.56 b	1322.80 b	2209.36 b	9.50 b	16.75 a	35.45 a	25.50 a	45.43 a	5.1 a
HighRH × light	770.38 c	1535.87 a	2306.25 b	10.10 ab	18.15 a	23.90 b	20.70 b	36.09 b	4.25 b
LowRH × dark	1040.94 a	1513.46 a	2554.40 a	10.60 a	16.50 a	15.25 c	16.70 c	34.71 b	2.9 c
HighRH × dark	863.92 bc	1572.69 a	2436.62 a	10.06 ab	17.50 a	23.65 b	15.35 c	18.99 c	3.25 c
Significance									
RH	***	***	NS	NS	NS	NS	***	***	NS
LR	**	***	***	*	NS	***	***	***	***
RH × LR	*	**	*	*	NS	***	*	*	**

**Table 3:** Analysis of variance and means comparison for functional metabolites and antioxidant activity of mung bean sprouts the four different combinations of RH and LR (LowRH-L; LowRH-D; HighRH-L; HighRH-D). Data are expressed on a fresh weight basis. Mean values and statistical significance are shown. NS, *; **, *** Non significant or significant at *p* ≤ 0.05, 0.01, and 0.001, respectively. Different letters within each column indicate significant differences according to Duncan’s multiple-range test (*p* ≤ 0.05).

Treatments	Anthocyanins (mg g^−1^)	Polyphenols (mg GAE mg^−1^)	Antioxidant Activity (µmol TE/mg^−1^)	Soluble Sugars (µmol (glucose) Eq. g^−1^)	Starch (µmol (glucose) Eq. g^−1^)
Relative humidity (RH)					
Low	0.85 a	0.022 a	1.01 a	0.24 b	0.047 a
High	0.68 b	0.013 b	0.57 b	0.53 a	0.027 b
Light regime (LR)					
Light	0.93 a	0.026 a	1.02 a	0.33 b	0.046 a
Dark	0.60 b	0.011 b	0.55 b	0.44 a	0.028 b
Significance					
RH	***	***	***	***	*
LR	***	***	***	**	*
RH × LR	*	*	***	**	*
